# PUblications Metadata Augmentation (PUMA) pipeline

**DOI:** 10.12688/f1000research.25484.2

**Published:** 2021-04-12

**Authors:** Oliver W. Butters, Rebecca C. Wilson, Hugh Garner, Thomas W. Y. Burton

**Affiliations:** 1 Department of Public Health, Policy and Systems, University of Liverpool, Liverpool, UK; 2 Population Health Sciences Institute, Newcastle University, Newcastle upon Tyne, UK; 3 Social and Community Medicine, University of Bristol, Bristol, UK; 4 Department of Computer Science, University of Oxford, Oxford, UK

**Keywords:** Longitudinal birth cohort, Bibliography, Bibliometrics, ALSPAC

## Abstract

Cohort studies collect, generate and distribute data over long periods of time – often over the lifecourse of their participants. It is common for these studies to host a list of publications (which can number many thousands) on their website to demonstrate the impact of the study and facilitate the search of existing research to which the study data has contributed. The ability to search and explore these publication lists varies greatly between studies.

We believe a lack of rich search and exploration functionality of study publications is a barrier to entry for new or prospective users of a study’s data, since it may be difficult to find and evaluate previous work in a given area. These lists of publications are also typically manually curated, resulting in a lack of rich metadata to analyse, making bibliometric analysis difficult.

We present here a software pipeline that aggregates metadata from a variety of third-party providers to power a web based search and exploration tool for lists of publications. Alongside core publication metadata (i.e. author lists, keywords etc.), we include geocoding of first authors and citation counts in our pipeline. This allows a characterisation of a study as a whole based on common locations of authors, frequency of keywords, citation profile etc. This enriched publications metadata can be useful for generating study impact metrics and web-based graphics for public dissemination. In addition, the pipeline produces a research data set for bibliometric analysis or social studies of science. We use a previously published list of publications from a cohort study as an exemplar input data set to show the output and utility of the pipeline here.

## Introduction

Cohort studies collect, generate and distribute huge amounts of longitudinal data for health, social and economic research based on a defined group of people over an extended period of time (often many years). Birth cohort studies begin at birth (or sometimes before) and often continue over the course of their participants’ entire lifetime. The UK is home to many cohort studies and several birth cohort studies, including some that have been running for decades (e.g. the National Survey of Health and Development (NSHD), which started in 1946 ^1^). Many of these cohort studies are prospective and non-disease specific, which necessitates broad data collection and results in a wide variety of research areas.

Typically, researchers can apply for, and access, these data sets once various relevant governance conditions have been met ^2^.

Cohort studies often keep track of the publications that have arisen from the data they have given to researchers to highlight the research carried out with their data, for monitoring purposes and to report back to their funder(s). These lists of publications often comprise of 1000s of items and the length of these lists are sometimes used as a crude metric of the impact of the study. It is common for studies to present these lists of publications on their websites as a means for interested parties to explore the outputs from their data.

We consider the lack of a comprehensive publication search and exploration facility a barrier to entry for researchers unfamiliar with studies that have large numbers of publications. When presented with potentially thousands of publications it can be difficult to find existing publications in a given research area, and when relevant publications have been found, lack of usage information (e.g. in the form of download statistics or citation counts) can make it difficult to prioritise reading lists. It is possible that some potential users of a study’s data fall at this first hurdle and do not proceed with an application for access to the data the study holds. This could have an effect on the overall impact of the study (since less new research is done) and in the case of studies that charge for data, it will have a direct financial implication. A similar scenario may occur if a researcher doesn’t find a relevant publication and applies for data to carry out a project that has already been conducted.

In addition to the difficulty of searching and exploring publications, lack of good metadata makes it impossible to do bibliometric ^1^ analysis on a study’s publications. This means questions such as ’where are all the first authors based?’ or ’are there trends in subject areas over time?’ can only be addressed either anecdotally, or with significant manual input (see e.g. 3).

The purpose of this work is to address the difficulty (or impossibility) of searching for publications and doing bibliometric analysis on studies which have a large number of publications. This is addressed by the development of an open source software pipeline (PUMA - **PU**blications **M**etadata **A**ugmentation pipeline) which takes a list of publications and augments it with metadata from a selection of third-party metadata providers. This augmented metadata set has two distinct uses: 1) enabling bibliometric analysis and 2) providing a web based searching and exploration tool of study publications. Examples of the potential bibliometric analyses possible with this augmented metadata include: calculating the total number of citations that publications based on a study’s data has generated, characterising a study based on the keywords of its publications, highlighting the geographic or institutional distribution of first authors, the variety of authors, assessing which journals are published in most frequently, how each of these metrics is changing over time, as well as other uses. We demonstrate some of these bibliometric uses and a web based exploration tool based on the augmented metadata set provided by PUMA in this article.

## Background

### Existing tools

There are several well established bibliography management tools in which users can manually curate their own bibliographies and easily use them to add formatted references to their written work (see https://en.wikipedia.org/wiki/Comparison_of_reference_management_software for a reasonable list). These include proprietary tools such as EndNote and Mendeley, as well as open source tools like Zotero. A common feature among them is to automatically incorporate available publication metadata from an external source (such as Web of Science, Scopus, CrossRef and others) into each bibliographic item. The wide variety and differing levels of completeness of available metadata means that typically a core set of fields are used. Also, static fields tend to be used in the tools, so an author list is common but a citation count is not. These subsets of all available metadata can typically be exported from the various tools in a variety of formats (e.g. BibTeX, RIS). There is little focus on gaining insight from the bibliographies in these software packages beyond grouping by keywords/themes.

The big three bibliometric metadata hubs (Web of Science, Google Scholar and Scopus) all have web based accounts which allow the curation of lists of journal articles and keeps track of the number of citations each article has. They also offer some basic citation analytics such as h-indexes and i10-indexes.

The focus of these bibliographic tools (both the online hubs and the software) is for an individual’s own published works, or an individual’s collection of publications which they may want to reference later on. Inbuilt to most of the tools is an automatic publication suggestion mechanism which uses the metadata of existing publications to suggest other publications based on common attributes (e.g. similar author lists or keywords).

There are several other tools which focus on specific visualisation or analytics of existing metadata sets. SurVis ^4^ creates an interactive web based exploration tool based on a static set of BibTeX metadata files. This allows filtering by author or keywords that exist in the static metadata files.

Network analysis of authors, subjects, journals, keywords and citations is another area of development, with tools such as CiteWiz ^5^, PivotSlice ^6^ and VOS Viewer ^7^ featuring analysis and visualistion of clusters, trends over time and in depth querying mechanisms.

These bibliography management, visualisation and analysis tools variously allow the curation of bibliographies, assist in finding similar articles, and give some insight to static metadata. No single existing tool gives easy access to aggregated and processed non-static metadata from a variety of sources to enable both in depth bibliographic study as well as providing an easy to use (potentially public facing) mechanism to explore publication metadata of a long running study.

### CLOSER studies

The CLOSER (Cohort & Longitudinal Studies Enhancement Resources) consortium ( https://www.closer.ac.uk) comprises 19 UK cohort studies and is used here as an illustration of typical cohort studies. Almost all of the CLOSER studies hold a list of their publications on their respective public facing websites. The specific purpose, functionality and user interface of these lists varies from study to study. Some studies have publications lists that are comprised exclusively of peer-reviewed journal articles, others have a much broader remit and include a variety of other written outputs, e.g. books, reports, conference proceedings and media examples. The way this data is presented varies greatly, ranging from downloadable static PDF files, through static lists on web pages split by year, to interactive web pages.

Where publications are listed on a static page the only way to search is by doing a browser-based free text search on the rendered text available on the page. Where only a subset of publications is shown (e.g. if it is split by year), or where rich metadata is missing (e.g. if no keyword or abstract text is available/rendered), it is difficult or impossible to search for given terms.

Some of the studies have a web form which allows a free text search on a database across author, journal, title and abstract text, split by year. One study (Understanding Society) has an advanced searching capability letting users search on author, subject, article type, as well as free text searching on title and abstract. None of the CLOSER studies have any kind of metrics kept alongside their publication lists, e.g. citations.

### Persistent identifiers

Modern academic journal articles are typically assigned persistent identifiers when they are published. The aim of these is to give a consistent and long-lasting mechanism to refer to them. Often a journal will assign a unique journal-specific identifier to an article which resolves to the article on the journal’s website. In addition to this, a Digital Object Identifier (DOI) is usually assigned. DOIs are the *de facto* persistent identifier used across the academic journal publishing sector.

DOI resolving services exist to refer users (human and machine) to the relevant journal web page. These resolving services also host a wealth of metadata themselves. The service used to resolve DOIs in this work is the canonical resolver: doi.org (see e.g. the DOI data model - https://www.doi.org/doi_handbook/4_Data_Model.html). 

In addition to doi.org there exist other resolving and metadata services that are domain-specific. These may have more in depth and domain specific metadata beyond that offered by the general data model provided by doi.org.

A lot of the publications generated from cohort study data are further indexed by the National Center for Biotechnology Information (NCBI) PubMed. PubMed generates PubMed IDs (PMID), and provides a metadata resolving service ^8^. This offers extra metadata over and above that available from doi.org, although on a subset of all available publications.

## Methods

### Implementation

**Figure 1.**
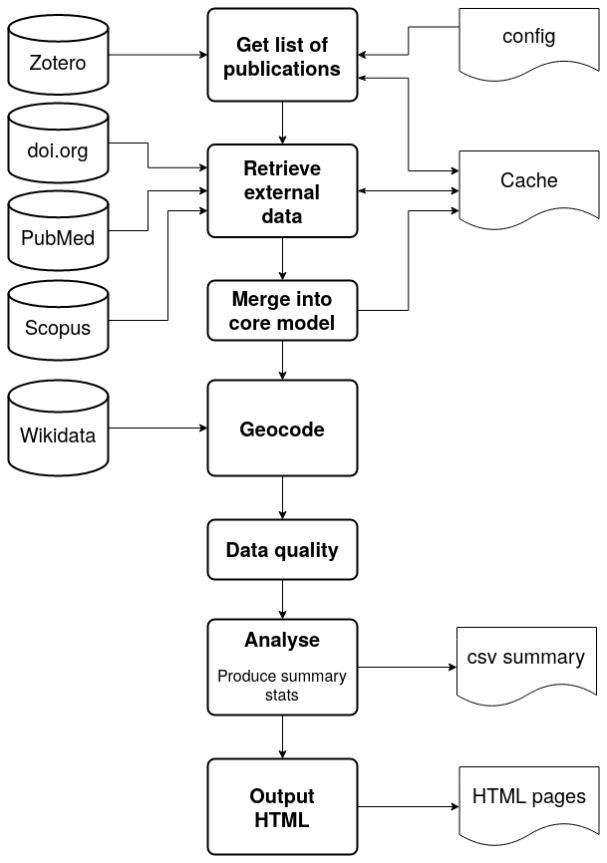
Overview of the PUMA pipeline. The left column shows the sources of data accessed via their APIs, the central column the stages the pipeline with the right column showing input and output of the pipeline.

PUMA is built as a pipeline of several discrete stages. The first stage retrieves a list of publications, then subsequent stages add and derive information for each publication in the list before passing it on to the next stage. The end goal of this augmentation stage is a consistent metadata object containing as many core metadata items as possible. This is achieved by first retrieving the list of publications from Zotero; adding metadata to it from doi.org, PubMed, and Scopus; geocoding the first author’s institute; and getting citation counts. The nature of the source metadata is such that fall-backs may be needed as metadata items may not be present in the first metadata source searched e.g. if the author list is not found in the PubMed metadata then the DOI metadata is queried next. Table 1 outlines this core metadata model and the source of each metadata item, along with fall-back sources. Once this metadata object has been built PUMA can then do some basic statistics and generate web pages to allow exploration and searching. The pipeline is explained in detail below, and shown in overview in Figure 1, where the stages of the pipeline (middle column) correspond to the sections below.

**Table 1.** Final metadata object. Tabular representation of the python dictionary used to store the metadata, the secondary column items are nested under the primary items where present. Metadata source key: Zr=Zotero raw, S=Scopus, D=doi.org, P=PubMed, Ze=’Extra’ field from Zotero, W=Wikidata, De=Derived. The metadata sources are used in the order they are displayed in the table (left to right), once a value has been found the subsequent sources are not queried.

Primary	Secondary	Source
IDs	DOI	Zr
	PMID	Ze
	Scopus	S
	Hash	De
	Zotero	Zr
Authors	Author list	P/D
	First author	Author list/Ze
	Affiliation	D/P/S/Ze
Location	Canonical institute	De
	Town	W
	Country	W
	Longitude	W
	Latitude	W
Date		P/D/S/Ze/Zr
Title		P/D/S/Zr
Abstract		P
Citations	Scopus citation count	S
Keywords	MeSH	P
	Other	P
Journal	Name	D/P
	Volume	D/P
	Issue	D/P

***Get list of publications: Zotero.*** Zotero is an online, free-to-use and open-source bibliography manager. It allows publications metadata to be grouped together into user defined libraries. Here we use it to hold the canonical list of unique publications for a given study. Zotero allows publications metadata to be entered manually (by filling in the fields by hand), semi-manually (by adding e.g. a DOI and it querying external sources), or programmatically using its API.

Once a publication’s metadata is stored in Zotero it can be updated as required. Data cleaning can be done in Zotero, and this is made easier by Zotero pulling in metadata from external sources. This can highlight, and enables easy fixing of, errors such as duplication of publications or where a DOI has been mistyped and points to an incorrect publication.

The PUMA pipeline begins by using the Zotero API (v3) to get the library for the study. This is presented as a series of JSON files (one per publication in Zotero), containing all the metadata held on a given publication. These are downloaded and cached locally by the pipeline.

While all of the metadata is downloaded from Zotero, the PUMA pipeline disregards most of it as it does not map to the final metadata object well. The most important fields used from the Zotero metadata are the DOI and PMID, since these are the identifiers used to query external metadata providers. DOI has a native field in Zotero, but PMID does not and is stored as a key-value pair in the Zotero ’extra’ field. There are a small number of fields where if the metadata is missing from doi.org and PubMed, then the metadata is used from Zotero. Where there is a direct match to the native Zotero data type (e.g. title) that is used, where there is not a match (e.g. Zotero doesn’t have an affiliation field for authors) then a key-value pair is used in the ’extra’ field. This is outlined in Table 1.

***Retrieve external data: doi.org.*** The pipeline then cycles through the list of publications, and where a DOI is present in the metadata from Zotero, it queries the doi.org API with it. If it is a valid DOI then doi.org will return a JSON file containing all the metadata it holds on this publication, which is cached locally.

***Retrieve external data: PubMed.*** If the Zotero metadata contains a PMID then the pipeline will then query the PubMed central API to get any extra metadata. The resulting XML file is cached for later use.

***Retrieve external data: Scopus.*** Scopus is then queried via its API. The query is first tried with a PMID, then if no value is found the query is repeated using the DOI as the identifier.

The use of Scopus data has some constraints on it depending on the context in which it is used. The most relevant condition here is that where citation counts are displayed on a website they must link back to the relevant publication in Scopus, and must be updated at least weekly ( https://dev.elsevier.com/tecdoc_attribution_ scopus.html).

***Merge into core model.*** As noted earlier, the metadata from doi.org and PubMed will contain different fields. Moreover, the same field may have different names in the two sources. In order to merge the metadata in a meaningful way we developed a mapping from each of the relevant fields to what we consider the local canonical version. In some cases, our mappings required several fall-backs, e.g. the date of a publication in PubMed has six different places that it could be specified. This is due to a combination of the PubMed schema changing over time, the completeness of the metadata when it is input into PubMed, and genuine different relevant dates e.g. date published online and date published in print.

The mapping is done into our core set of fields (see Table 1) for each metadata source. Our mapping process initially creates a simple metadata object based on the Zotero ID, DOI and PMID. Into this metadata object it then copies the relevant fields from the DOI, PubMed and Scopus metadata.

***Geocode.*** We assume the first author of the publication is the primary author, then we attempt to assign a canonical institute to them. This assignment is done by using a manually built lookup table which initially tries to use the email address of the first author, then if that fails, the postal address. In order to get consistent geographical information of a publication we take a university to be the smallest unit (i.e. two different departments at the same university will not be distinguished in the geocoding). The reason behind this is that there is very little consistency between publications on how department addresses are formatted. The same strategy is used for hospital departments and companies. Our definition of what a canonical institute name is is based on how it appears in wikidata ( https://www.wikidata.org). 

For the email address based matching we attempt to match exactly the domain part of the email address to a canonical institute (e.g. someone@ucl.ac.uk gets mapped to University College London). Email addresses that are generic or personal (e.g. someone@gmail.com) are ignored.

If there is no matching email address then we attempt to match the postal address. The lookup table has multiple entries for several organisations where authors use non-canonical names e.g. ’UCL’ and ’ University College London’ both map to University College London.

Since a publication list may go back many years, there may be institutes that no longer exist (perhaps having been renamed, merged with other institutes or shut down entirely). The lookup table therefore has several entries of now defunct institutes which are mapped to from email addresses and postal addresses.

Once we have the canonical name for an institute we use the wikidata SPARQL API to get the institute’s geolocation, town and country (wikidata properties: P625, P131 and P17, respectively). In some cases, the first author’s institute may be a large multinational or distributed organisation, in which case we use the headquarters location as defined on wikidata (property P159). If any of this data does not exist on wikidata we try to add it.

***Data quality.*** The nature of the manual curation of a list of publications can lead to some missing information and errors. This ranges from there not being any persistent identifiers present, to multiple copies of the same publication being present in different forms, e.g. a preprint and the final version. In order to address these data quality issues, we built an interface to assist further cleaning of the metadata, there are two main facets to this interface: highlighting issues and making fixing issues easier.

The interface consists of two HTML tables, the first displays the number of publications where metadata exists for each of the items in Table 1. The second table has a row for each publication and columns for the status of relevant attributes. Where a value of an attribute is useful in the data cleaning process it is displayed (e.g. DOI and PMID), where the presence of an attribute is more useful than its value (e.g. first author) then just an indication of its presence is given. Missing attributes are colour coded to make them easy to see, and the table can be sorted by value/presence of attributes. Where, for example, a PMID is missing, the relevant table cell is coloured orange and there is a clickable link which queries PubMed for this publication based on its DOI or title. Similar approaches are available to find DOIs via PubMed and Scopus. Where this provides missing IDs (DOI or PMID) they can be added to Zotero and the pipeline rerun.

Some metadata may not be present in the external providers metadata for some publications-even with the correct DOIs and PMIDs in Zotero. In this case the metadata can be used directly from Zotero for a small number of fields as indicated in Table 1. As with the DOI and PMID case above, the missing metadata will be highlighted orange in the table, and once it has been added to Zotero the pipeline will need to be run again.

There are some derived metadata items that, if missing, will be highlighted in red; this indicates that a setting in the pipeline or a local configuration file is causing the problem. An example would be if a first author institute is found in the source metadata, but there is no matching entry in the institute look up file then a canonical institute cannot be set. The lookup file needs to be updated and the pipeline rerun in this case. A sample screen shot of this page is linked to in the output data in the data availability section.

***Analyse.*** The pipeline then does some simple processing of the metadata so it can be used for reporting and which feeds into the generated web pages (see below). It outputs (as a CSV file) the frequency of the authors (separately the full author list and first author only), the first author’s institute and the journal the publication appeared in.

The keywords, title and abstract text in a publication all serve to give an overview of the content. The keywords are sometimes from a controlled vocabulary, e.g. Medical Subject Headings (MeSH). The titles and abstracts having more free text offer the ability to be more descriptive. From a searching perspective the greater freedom with abstracts makes them more searchable/findable ^9^. To derive some meaning from all of the available text in all of the publications from a study, the pipeline calculates the frequency of each word in the keywords, titles, and abstracts. To process the text it converts all text, to lower case and removes all punctuation. It then takes out the name of the study, so in the use case below the exact phrase "Avon Longitudinal Study of Parents and Children", and variations of it, are removed, but individual components are kept if they were used outside of that context e.g. if ’parents’ is used in a different sentence. Then common words such as *the*, *and* etc are disregarded. Then the Python Natural Language Tool Kit ^10^ is used to lemmatize each word into its base component. With this clean set of words, the pipeline then calculates the frequency of each. It also does this broken down by year, so it is possible to see how trends in research areas change over time in a long running study.

See the data availability section for examples of these outputs.

***Output HTML.*** The pipeline generates static HTML pages which allow the search and exploration of the augmented metadata sets. These pages include filtering by year and by keywords, and visualisations of some of the metadata. Where a list of publications is displayed (e.g. after filtering on a keyword) the citation count of each publication is displayed. This helps users to quickly identify impactful publications.

The static HTML pages are completely encapsulated, meaning that they can be viewed without the need for a web server. As such, PUMA can run locally to generate the data and statistics, and then the HTML files used to explore it.

**Figure 2.**
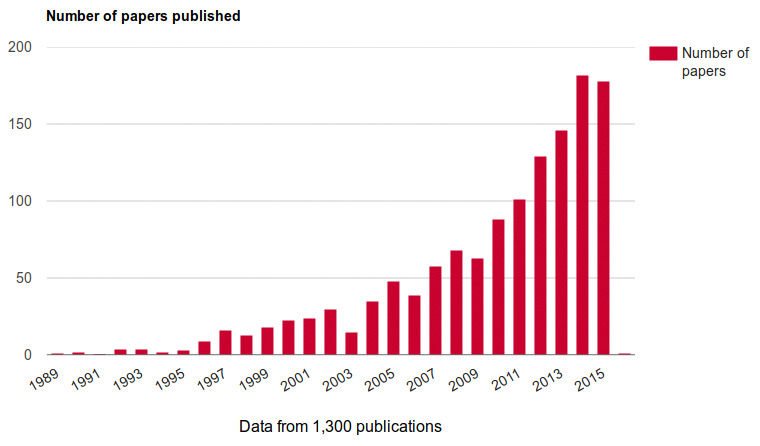
Number of publications per year in ALSPAC.

**Figure 3.**
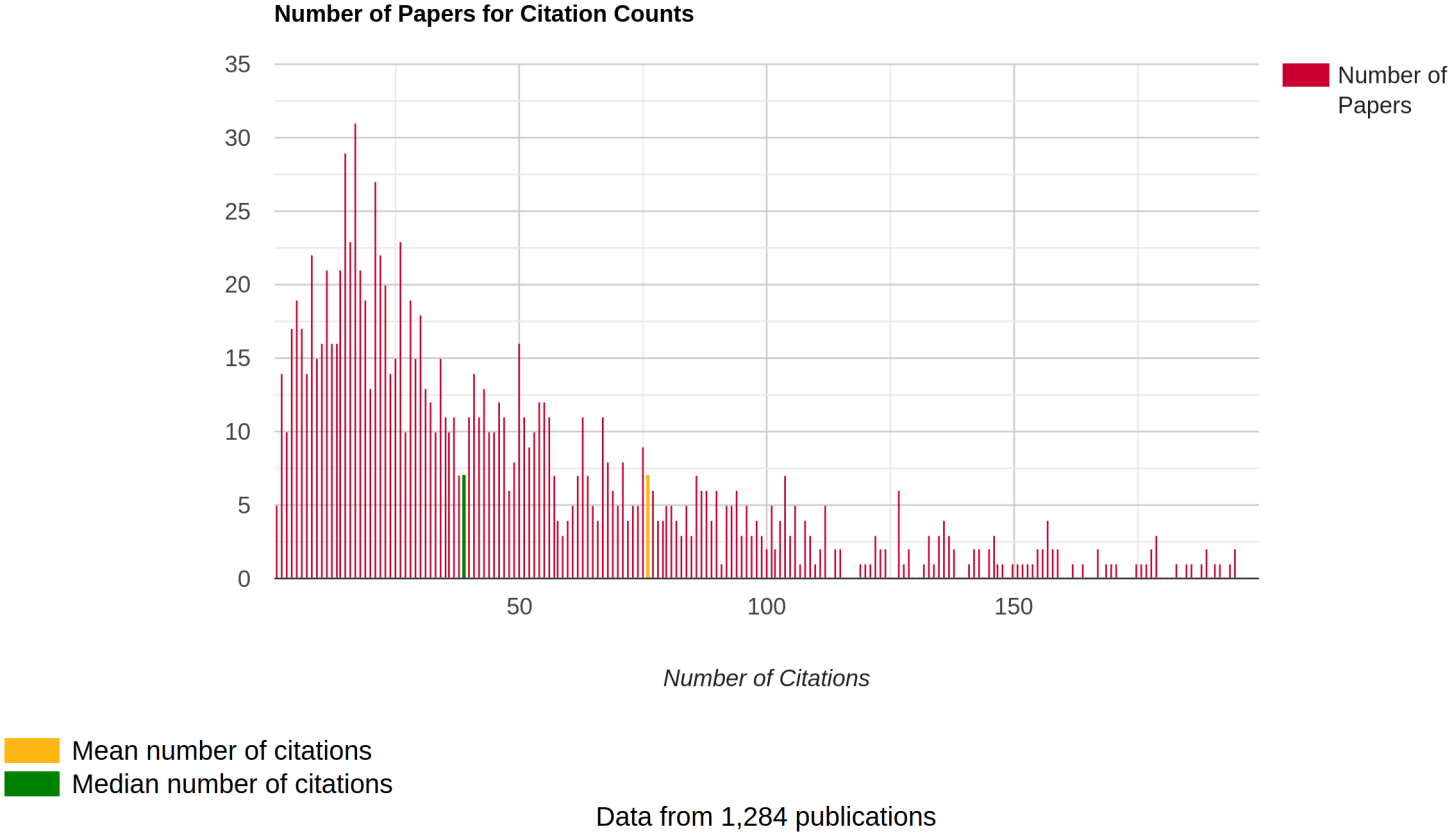
Citation count profile of ALSPAC publications as of 15/1/2021. The x-axis is truncated at 200 citations as there are a small number of publications disparately spread above this.

**Figure 4.**
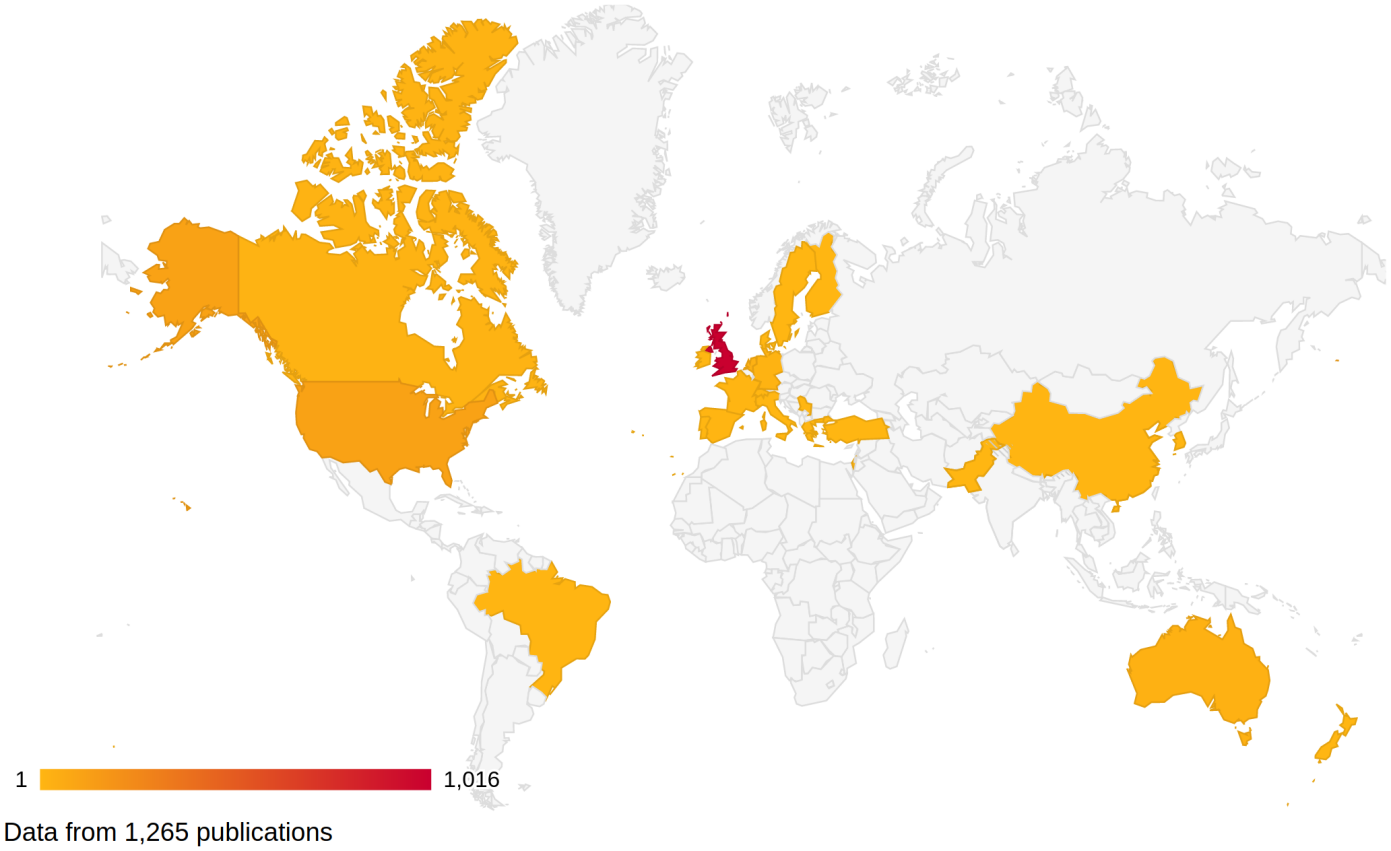
Choropleth map of first author countries in ALSPAC.

Figure 2, Figure 3 and Figure 4 show example plots taken from the generated web pages for the use case outlined below. Full screenshots are linked to in the outputs section below, with live versions available at https://ollybutters.github.io/puma/alspac/.

### Operation

The pipeline is written in Python 3 and is available from GitHub ( https://github.com/OllyButters/puma). Some prerequisite Python libraries are required to run the PUMA pipeline, these are described on the wiki at https://github.com/OllyButters/puma/wiki and in the requirements.txt file in the source folder.

The behaviour of the pipeline (which API keys to use, date ranges, colour schemes, caching behaviour etc) is controlled by a configuration file. A sample configuration file is available in the *config* directory, with guidance on how to populate it at https://github.com/OllyButters/puma/wiki/Configuration. The pipeline has been developed in linux environment, and can be run from the command line by calling the *papers.py* file in the source directory. This assumes a *config.ini* file in the *config* directory, if a different file name is used then it can be specified when running the pipeline with the config option: *papers.py –config config-file-name.ini*. We have also run PUMA in a Windows 10 environment with a minimum of python 3.6.

The pipeline is designed so that it can be rerun regularly, running as a regular CRON job for example. Metadata is cached locally wherever possible, making subsequent pipeline runs much quicker after the initial run. This is possible as the metadata from doi.org and PubMed is very stable, so changes are rare to an individual publication’s metadata. The citation counts are cached for as long as is specified in the configuration file, being updated as required.

The pipeline is designed so that it can be rerun regularly, running as a regular CRON job for example. Metadata is cached locally wherever possible, making subsequent pipeline runs much quicker after the initial run. This is possible as the metadata from doi.org and PubMed is very stable, so changes are rare to an individual publication’s metadata. The citation counts are cached for as long as is specified in the configuration file, being updated as required.

Given the command line nature of running the pipeline and the encapsulated output HTML pages, we foresee two main scenarios where PUMA is likely to run: firstly, on a public facing website run by a cohort study where the primary objective is to enable relevant publications to be searched and explored. The second scenario is where a researcher may run the pipeline on their desktop in order to collate metadata to do a bibliographic analysis of a study. In both of these scenarios there is a relatively high degree of technical skills required – being able to install, configure and run python programs, with the first scenario also requiring web hosting knowledge.

## Use case

While the intention of this article is to describe the pipeline and the work flow developed to ingest metadata into it we also show some example outputs of the pipeline with minimal interpretation.

### Exemplar publication list

In this work we make use of an exemplar list of publications from the Avon Longitudinal Study of Parents and Children (ALSPAC - https://www.bristol.ac.uk/alspac). ALSPAC reports to have over 2000 publications as of April 2019 ( http://www.bristol.ac.uk/alspac/news/2019/bristol-families-co90s.html). For this work we use the cleaned BibTeX list of ALSPAC publications ^11^ described in 12. For a general overview of ALSPAC see 13.

The ALSPAC began in 1990 and as a consequence of this their publications are relatively modern and there is a good coverage of DOIs. The nature of the research done with ALSPAC data is largely biomedical, which gives a high proportion of publications with PMIDs. See Table 2 for a summary of the coverage of the source metadata.

**Table 2.** Source metadata coverage.

Date range	1989–2015
Publication count	1300
DOIs	1260
PMIDs	1240
At least one of DOI or PMID	1293

### Running the pipeline

We imported the ALSPAC BibTeX data into a new collection in a new group library in Zotero, giving the coverage of fields as outlined in 12. The PUMA pipeline works best with at least one of DOI and PMID for each publication, coverage of these fields in the source metadata is outlined in Table 2. See *Underlying data* for a list of the references used ^11^.

For this initial metadata set the pipeline achieved the augmented metadata coverage outlined in Table 3. Where there are gaps in this metadata it is mostly due to actual missing metadata in the source systems, however the incompleteness in the geocoded metadata is due to a combination of authors using a consortium name as their affiliation, or the metadata containing only a fragment of their address. These could easily be manually addressed with the ’extra’ field in Zotero, however since the purpose of this article is to outline the PUMA pipeline and not to strive for a 100% coverage of the metadata, we have not added any ’extra’ metadata to Zotero.

**Table 3.** Counts of completeness of the augmented metadata fields. The values are taken from the coverage report web page generated by the pipeline. A screenshot of this page is available in the output data (see data availability).

Publication count	1300
First author name	1288
Raw first author institute	1279
Derived institute	1271
Derived geolocation	1268
Year published	1300
Publication title	1300
Abstract	1207
Scopus citations record	1284
Keywords (MeSH)	1205
Journal Name	1293

The initial run of the pipeline took 2.5 hours, with a subsequent rerunning taking approximately two minutes, highlighting the amount of time spent downloading data from remote sources and the importance of caching the data.

### Number of publications per year

Figure 2 shows the number of publications published per year for ALSPAC from 1989 to 2015. This is the most basic information from the pipeline, and is already information that is easily available to the studies.

### Simple citation statistics

Table 4 shows some basic study level citation calculations. As noted above, the incompleteness of the metadata will impact the numbers here, specifically, ALSPAC has 98% Scopus coverage, meaning all the citation-based numbers in Table 4 will likely be slightly under-reported. Further to this, Scopus will not hold all publications which themselves cite these publications (see limitations below), so the values in Table 4 are likely under-reported more so. Even with this under-reporting, the citations indicate that the publications which arise out of the ALSPAC data are themselves regularly cited.

**Table 4.** Study level citation statistics from Scopus as of 15/1/2021. The values are taken from the metrics web page generated by the pipeline. A screenshot of this page is available in the output data (see data availability).

Number of publications	1300
Number with citation data	1284
Total citation count	97,537
h-index	141
c100-index	226
Mean citations per publication	76
Median citation count	39

Figure 3 shows the profile of citation counts for ALSPAC publications with a citation count less than 200.

### Geolocation

Using the geolocation data generated from the pipeline we can plot a choropleth map of the countries that first authors are based in. Figure 4 shows the first authors location for 98% of the publications. Again, this is affected by the coverage of the source metadata.

The plot indicates that the majority of first authors are based in the UK. This is perhaps expected due to the nature of the research carried out with cohort study data.

### Keywords, titles and abstracts

Table 5 shows the result of a frequency analysis of lemmatized keywords, title text and abstract text of all of the publications that have relevant metadata. These most frequent words correspond with the overall nature of ALSPAC – i.e. a birth cohort study which has followed its participants over a long period of time.

**Table 5.** Frequency of top ten lemmatized words used in keywords, titles and abstract text from the ALSPAC publications. The full list of words as output by the pipeline is available in the output data (see data availability). The numbers in parentheses are the count.

Keywords	Title	Abstract
study (1513)	study (357)	child (2517)
child (1284)	child (291)	age (2034)
human (1257)	cohort (259)	association (1905)
female (1050)	childhood (220)	associated (1696)
male (859)	association (220)	study (1675)
factor (720)	birth (146)	year (1553)
infant (568)	age (129)	risk (1142)
longitudinal (562)	risk (128)	maternal (1120)
pregnancy (470)	maternal (122)	ci (915)
adolescent (470)	associated (117)	cohort (904)

These frequencies can be broken down by year to show study changes over time. There are some obvious single word changes in the metadata, e.g. the use of the word *puberty* in the abstracts increases when the children reach their teens. It is important to emphasise that this represents the years of the *publications* about puberty and does not represent when the participants were going through puberty themselves. Similarly, as new technologies and techniques were developed and used they start to appear more frequently, e.g. ‘genetic’ is mentioned for the first time in 1998 and 100 times in 2014. There are almost 9000 unique words across the abstracts which PUMA has calculated the frequencies of. This data can serve as the starting point for a more in-depth thematic analysis of the publications which has not been possible before, and is beyond the scope of this article. 

## Discussion

We have highlighted the potential difficulty of analysing and exploring publications in studies with large numbers of publications and how this could have an impact on use of a study’s resource. We have shown here the development of a software pipeline - PUMA - which can take a list of publications and aggregate external sources of metadata to automatically generate datasets ready for bibliometric analysis and standalone web pages ready for local exploration or for public facing web hosting.

While the exemplar publications list used here is a U.K. birth cohort, this pipeline could be applied to almost any research study that has a list of publications with a rich set of persistent identifiers, particularly in the biomedical domain.

One of the difficulties in developing the pipeline was the lack of a good list of publications, with gaps in the metadata for the list of publications leading to issues with the pipeline being able to process data. As the pipeline matured we put in place more tools to find, and eventually help fix, the gaps in the raw metadata.

### Limitations

One limitation to this work, which is difficult to address, is the completeness of the source list of publications. It is common for cohort studies to ask researchers to inform them when they publish their research based on the study’s data. This request is not always complied with, so the source lists of publications are prone to being incomplete. This will have an impact on the insights the PUMA pipeline can generate, with some aspects just under-reporting (e.g. the total citation count) while others may give a misleading picture if there is a systematic reason for the missing publications (e.g. the frequency of keywords in a study will be misleading if all publications from a field are missing).

One of the key assumptions we have made is that the first author is the primary author for the publication. This does vary across different scientific disciplines - it may be that the first author is the one who did the bulk of the work, or that they wrote up the majority of the publication, or they just appear first alphabetically. While this will not have an effect on the publication-level statistics (e.g. how many citations it has), it may have an effect on where we have assigned a geographic location.

Linking on author name is also problematic when multiple authors have the same name, or where there are multiple spellings of a given name. This can occur where names have been converted to e.g. ASCII on their way into metadata records. Another instance is where a name is sometimes hyphenated and others not (in this exemplar data set there exists entries for Davey Smith and Davey-Smith).

It is important not to place too much emphasis on citations and to not treat them as the definitive value. While it is easy to count how many publications are cited in a given publication, it is difficult to establish the inverse - i.e. how many publications in all the literature cite a given publication. This is due to the completeness of the source literature which is used to calculate the incoming citations, which means that different providers of citation counts will likely give different answers (see 14 and 15).

The command line interface to run the pipeline may limit the potential userbase of PUMA who may want to use it for bibliometric research. A more interactive graphical user interface may help address this if this does prove to be the case in the future.

## Future work

The modular nature of the pipeline means that it is straightforward to add different data sources. One source that we plan to add is Altmetric, which tracks mentions of publications in the media (including social media) and links these back to a DOI. We also plan to link directly with Crossref (using their API) to pull in a richer set of metadata.

Some of the modern PubMed metadata, and a lot of the Crossref metadata, include information on grants (increasingly with a grant reference code). This would allow us to investigate who the major funders of users of the data are.

## Data availability

### Underlying data

The list of publications in the use case are available from Zenodo: ALSPAC peer reviewed publications 1989–2015. http://doi.org/10.5281/zenodo.2276785 ^11^.

All other metadata is pulled in from external APIs at run time.

### Outputs

Full page screenshots of a sample of the generated web pages, as well as the output CSV files are available at http://doi.org/10.5281/zenodo.4545742

Data are available under the terms of the Creative Commons Attribution 4.0 International license (CC-BY 4.0).

## Software availability

**Source code available from:** https://github.com/OllyButters/puma.

**Archived source code at time of publication:** http://doi.org/10.5281/zenodo.3971102 ^16^.

**License:** GNU General Public License v3.0.

## Footnotes

^1^ Bibliometrics is the use of statistical methods to analyse books, articles and other publications.
