# Supplementary material for: PUblications Metadata Augmentation (PUMA) pipeline
Source: F1000Res. 2021 Apr 12;9:1095. Originally published 2020 Sep 4. [Version 2] doi: 10.12688/f1000research.25484.2 (PMC8108552; doi:10.12688/f1000research.25484.2)
Supplement: Peer review history

# Peer review: Reviewer response for version 2

**DOI:** 10.5256/f1000research.55033.r83114

**Published:** 2021-05-05

**Authors:** Leslie McIntosh

**Affiliations:** 1 Ripeta, Saint Louis, MO, USA

The authors addressed all my concerns and comments. I did not attempt to rerun the software for this review, however, it did run for the first review. Great work!

Are the conclusions about the tool and its performance adequately supported by the findings presented in the article?

Yes

Is the rationale for developing the new software tool clearly explained?

Yes

Is the description of the software tool technically sound?

Yes

Are sufficient details of the code, methods and analysis (if applicable) provided to allow replication of the software development and its use by others?

Yes

Is sufficient information provided to allow interpretation of the expected output datasets and any results generated using the tool?

Partly

Reviewer Expertise:

Informatics, bibliometrics, scientometrics, biomedical informatics

I confirm that I have read this submission and believe that I have an appropriate level of expertise to confirm that it is of an acceptable scientific standard.

# Peer review: Reviewer response for version 2

**DOI:** 10.5256/f1000research.55033.r83113

**Published:** 2021-04-29

**Authors:** Meena Khatwa

**Affiliations:** 1 Evidence for Policy and Practice Information and Co-ordinating Centre, UCL Institute of Education, University College London, London, UK

I have read through the recent revisions based on my questions and now approve the article.

Are the conclusions about the tool and its performance adequately supported by the findings presented in the article?

Partly

Is the rationale for developing the new software tool clearly explained?

Yes

Is the description of the software tool technically sound?

Yes

Are sufficient details of the code, methods and analysis (if applicable) provided to allow replication of the software development and its use by others?

Partly

Is sufficient information provided to allow interpretation of the expected output datasets and any results generated using the tool?

Partly

Reviewer Expertise:

NA

I confirm that I have read this submission and believe that I have an appropriate level of expertise to confirm that it is of an acceptable scientific standard.

# Peer review: Reviewer response for version 1

**DOI:** 10.5256/f1000research.28123.r70885

**Published:** 2020-12-16

**Authors:** Meena Khatwa

**Affiliations:** 1 Evidence for Policy and Practice Information and Co-ordinating Centre, UCL Institute of Education, University College London, London, UK

**Overall comments**

 This was a very interesting article on finding ways to find a user-friendly way to source and manage data.

 As a systematic reviewer, the processes used are not so dissimilar to how we scope and search for evidence. 

 On the whole I found the paper engaging and have only minor suggestions and comments to make. I am a qualitative research who specialised in qualitative evidence synthesis and thematic analysis. Therefore I positioned myself as someone who as a first time researcher would be interested in applying this method. 

**Abstract**

 Clear and concise but I would have made it more explicit this was project to trial/test PUma.

**Introduction**

 I understand this project was primarily funded by CLOSER; " *This project was primarily funded by CLOSER, whose mission is to maximise the use, value and impact of longitudinal studies. CLOSER is funded by the Economic and Social Research Council (ESRC) and Medical Research Council (MRC) (grant)"* hence using their data, as you state as an ‘illustration’ (p 3). It would have useful to have compared to another data set to have avoided any conflict of interest or biasness.

*“Some studies have a web form which allows a free text search on a database across author, journal, title and abstract text, split by year. One study has an advanced searching capability letting users search on author, subject, article type, as well as free text searching on title, abstract etc. None of the CLOSER studies have any kind of metrics kept alongside their publication lists, e.g. citations”.* (p3)

 This paragraph needs to make more clarity as it is unclear, what do you mean by one study and what is that study. The information is a bit vague.

*“We consider the lack of a comprehensive publication search and exploration facility a barrier to entry for researchers unfamiliar with a study. When presented with potentially thousands of publications it can be difficult to find existing publications in a given research area, and when relevant publications have been found, lack of usage information (e.g. in the form of download statistics, citation counts etc) can make it difficult to prioritise”* pg. three

 There is a disconnect between this paragraph and the one before – clearer connections between the arguments and rationale here how have the CLOSER studies led to this rationale in this paragraph.

 For first time users – it would be helpful to define what you mean by, **“bibliometric”,** as it used quite frequently in the paper.

**Exemplar Publication list (p4)**

 You state here the exemplar list of publications considered in this project is from the Avon Longitudinal Study of Parents and Children (ALSPAC). It would really be helpful from the outset if the author could be clear that this was a project undertaken to test the PUMA pipeline there seems to be a switch from *study to project* – to maintain consistency perhaps refer to one description.

**Methods**

 Page 7 - unclear by what you mean by **‘Use Case’** - what does this mean?

 I found the diagram and definition of each stage very helpful in regards to the process undertaken.

**Discussion**

 It would be really helpful to know how long it took to use PUma when trialling it for this project. Where there any technical glitches? How user friendly it is, particularly in regards to accessibility (especially for students or first time researchers)?

**Future work**

 If this is to be utilised by others what will be available as a resource guide for those who want to use it (e.g. training etc.)?

**Headings and signposting**

 There seem to be three types of headings being used to signpost different sections. At times I found this confusing as the main heading and subheading seem to be same font size and weight (e.g. see discussion the limitations underneath then you have sub subheadings in italics).

Are the conclusions about the tool and its performance adequately supported by the findings presented in the article?

Partly

Is the rationale for developing the new software tool clearly explained?

Yes

Is the description of the software tool technically sound?

Yes

Are sufficient details of the code, methods and analysis (if applicable) provided to allow replication of the software development and its use by others?

Partly

Is sufficient information provided to allow interpretation of the expected output datasets and any results generated using the tool?

Partly

Reviewer Expertise:

Primary Qualitative research, systematic reviews in evidence base policy in public health. Specialist in stakeholder engagement, qualitative evidence synthesis and thematic analysis. Lecture on MSc module : Introduction to Qualitative Methods.

I confirm that I have read this submission and believe that I have an appropriate level of expertise to confirm that it is of an acceptable scientific standard, however I have significant reservations, as outlined above.

# Response

**Published:** 2021-02-26

**Authors:** Olly Butters

**Affiliations:** University of Liverpool, UK

Thank you for taking the time to review this paper, and we’re very happy to hear you found it interesting! I think we have addressed all of your comments in the text. Below we itemise each one and how we have addressed it. 

**Abstract**

 Added text to be explicit about this being a test of PUMA using a list of publications from a cohort study. 

**Introduction **

- Comparing to another dataset – we found that the amount of work required to generate a clean list of publications was high (see the ALSAPC data paper referenced here). For now, we just wanted to show what the pipeline is. Our plan is to do as you suggest in the future and run the pipeline on a selection of different studies so that we can look in more detail at the differences between them.  
- Cohort study web form searching – tidied up language, and updated text to reflect CLOSER’s increased membership. 

- Restructured the introduction to make this clearer. 
- Added a bibliometrics definition 

**Exemplar publication list **

- Moved this section to the use case section and made it clearer throughout that this data is to show the pipeline working. 
- Tidied use of project/study throughout. 

**Methods**

 Use case - The term use case comes from the F1000 software tool article structure and it's just meant to show an example of the pipeline running.

**Discussion** 

- Outlined in the operation section that it is a rather technical program to run, and also mentioned in limitations section. Added the time it took to run in use case section. 
- Added discussion point about its development. 

**Future work**

 Currently, I'd expect the main users to be at the more technical/development end. The documentation on the wiki covers all aspects of how to run it and was sufficient for Leslie McIntosh to install and replicate the analysis. It would be interesting to partner with some bibliometric analysis researchers to see where the software and training needs to go next, but that will require more funding.

**Headings**

 Tidied up use of headings to make it easier to follow.

# Peer review: Reviewer response for version 1

**DOI:** 10.5256/f1000research.28123.r72761

**Published:** 2020-11-26

**Authors:** Leslie McIntosh

**Affiliations:** 1 Ripeta, Saint Louis, MO, USA

**Overall Comments**

 Improving the search capabilities across studies based on research manuscripts is challenging and important. The authors have built a pipeline to improve the capture of metadata, thus addressing this challenge. The links to the data and software work. Moreover, I replicated the study with the available data, code, and instructions without contacting the authors. While there are many comments below, the majority pertain to improving the article readability. Great work on building this pipeline.

**Open Science**

 This part of the review focuses on the transparency of the work looking for quality indicators that are important for a good manuscript. While all of these elements are not necessarily required by the publisher, the presence and clarity of them make your work more transparent.

Authors

 One author has an ORCID listed while the others do not. It is best practice to have unique, persistent author identifiers such as an ORCID for each author. 

Study Objective

 Your Text from the Introduction: *This article describes an open source software pipeline (PUMA -PUblications Metadata Augmentation pipeline) which takes a list of publications and augments it with metadata from a selection of third-party metadata providers.*

 Comment: This technically states what you are doing and is not a clear study objective. A clear, concise, study objective will help in reading and understanding the science (e.g., The purpose of building the software pipeline is to improve search capabilities across cohort studies through augmenting metadata.)  

Data Availability Statement

 The DAS is present and links to the Zenodo corpus of papers for the Use Case. You could also state that the other data presented is generated from the software/code.

Code Availability

 The code is available as stated in the Software Availability statement and is working as expected.

Funding Statement

 This is present with appropriate grant identifiers mentioned. I did not verify the grant IDs.

Ethics Statement

 When reviewing your manuscript, I could not find the ethical approval of the research. As this appears not to apply to this research, this is acceptable.

**Organisation and Miscellaneous**

 This section provides critiques based on the readability of the article. Where appropriate, I offer suggestions for making it more consumable to the reader.

Introduction

 The information is great in the introduction, but I found it difficult to read at first. Suggestions for improvement:

- The first through fifth paragraphs (ending  with “The search functionality… paragraph” seems out of place. Perhaps call out the description of the cohort study data just has you have other topics in the introduction (e.g., Existing Tools, Exemplar Publication List) and put it later (see next comment).
- The true introduction seems to start with the sixth paragraph “We consider the lack of a comprehensive publication…” until the ‘Existing Tools’ Section. This section succinctly describes the problem with searching across cohort publications and offers your study objective.

From the sentence beginning with “In this work we also make use of the persistent identifiers that the National Center for…” through the end of the next section appears to have truly started describing your Methods. 

Methods

1. The Implementation subsection is a bit challenging to read. It might make it more readable to add labels to Figure 1 so they can be referenced in each section describing it. There are invariably other possible solutions as well.
2. Describe Table 1 in more detail before it is referenced.
3. Figure 3 - the mean and median are hard to see with the yellow and green colours. (You don’t necessarily need to fix this in the paper but maybe in a future iteration of the software.)

Other 

- The keywords for the article should include something related to pipeline development and/or software.
- There are a number of grammatical errors throughout the article.
- Consider dropping ‘etc.’ when you are stating examples, especially when using ‘(e.g., )’

**Scientific Review**

 This section delves into the scientific question(s) presented in the article. I am specifically looking for an interesting scientific rationale for the study as well as supporting evidence to answer the research objective(s).****

Study Objective as stated in article****

*This article describes an open source software pipeline (PUMA -PUblications Metadata Augmentation pipeline) which takes a list of publications and augments it with metadata from a selection of third-party metadata providers. This augmented metadata set has two distinct use cases: 1) bibliometric analysis and 2) providing a web based searching and exploration tool.*

*Examples of the potential bibliometric analyses possible with this augmented metadata include: calculating the total number of citations a study has generated, characterising a study based on the keywords of its publications, highlighting the geographic or institutional distribution of first authors, the variety of authors, assessing which journals are published in most fre- quently, how each of these metrics is changing over time, as well as a variety of other uses. We demonstrate some of these bibliometric uses and a web based exploration tool based on the augmented metadata set provided by PUMA in this article.*

1. The software ran on my local machine and using the corpus suggested within Zotero, everything replicated. Great job!
  Working with Zotero does provide a few challenges, but there are notes on the GitHub wiki that walks through those challenges.
2. The counts provided Tables 2-5 come from a CSV file generated after running the code. It would be nice to have the files and code for the use case to be shared as well. This not only supports complete reproducibility, it also helps some readers understand the expected outputs without installing the software.
3. The Use Case is very nice to have in these types of projects, so I would suggest being clearer with it. What questions can you answer from these data that you could not before? Why is this important?
4. The Discussion should loop back to the purpose of the study and restate what you achieve through your work.

Are the conclusions about the tool and its performance adequately supported by the findings presented in the article?

Yes

Is the rationale for developing the new software tool clearly explained?

Yes

Is the description of the software tool technically sound?

Yes

Are sufficient details of the code, methods and analysis (if applicable) provided to allow replication of the software development and its use by others?

Yes

Is sufficient information provided to allow interpretation of the expected output datasets and any results generated using the tool?

Partly

Reviewer Expertise:

Informatics, bibliometrics, scientometrics, biomedical informatics

I confirm that I have read this submission and believe that I have an appropriate level of expertise to confirm that it is of an acceptable scientific standard, however I have significant reservations, as outlined above.

# Response

**Published:** 2021-02-26

**Authors:** Olly Butters

**Affiliations:** University of Liverpool, UK

Thank you for taking the time to read the paper and installing/running it! We think we have addressed all of your comments and have itemised this below.  

**Open Science**

 ORCIDs – All authors have now added ORCIDs. 

 Study objective – Updated the text as suggested. 

 DAS – added an output section with a link to Zenodo with all the output data and some screenshots. 

**Organisation and Miscellaneous**

 Introduction - Substantially restructured the introduction along the lines suggested here and in the other referee's comments. Some content moved into a separate background section and the use case data description moved to the use case section later on.  

 Method 

- Aligned the heading names in the implementation section to the middle column of the figure and highlighted this in the text. 
- Reworded how and where table 1 is referred to. 
- The colours on the plots are mostly configurable in the software, but I agree it is not easy to see that in the diagram. I have added the median value to the citation metrics table to help with this paper. 

Other 

- Keyword – Added ‘Software pipeline’ 
- Fixed typos and grammatical errors throughout. 
- Got rid of several ‘etc’  

**Scientific review**

 Glad you got it to run! 

 Table data:

- Table 2 – The counts come from manually looking at the raw data. 
- Table 3 – The metadata coverage table values are copied from the output HTML. I have added a screen shot in the Zenodo output data of this and it is signposted in the table caption.   
- Table 4 – The citation values are copied from the output HTML. I have added a screen shot in the Zenodo output data and this is signposted in the table caption. 
- Table 5 – Lemmatized word frequencies – Added the full list of lemmatized words to the Zenodo output data and signposted in the caption. 

Use case – I have updated the text to have a brief discussion about some of the derived data. 

 Discussion – updated the start of the discussion to be more of a summary.
